# Late presentation of critical upper limb ischemia caused by pseudarthrosis of the clavicle

**DOI:** 10.1590/1677-5449.009617

**Published:** 2018

**Authors:** Marcio Miyamotto, Lucas Vasconcelos Sanvido, Luan Facttore Brendolan, Amilton Cezar, Giana Caroline Strack Neves, Izara Castro de Souza, Ricardo César Rocha Moreira

**Affiliations:** 1 Pontifícia Universidade Católica do Paraná – PUC-PR, Hospital Universitário Cajuru – HUC, Serviço de Cirurgia Vascular e Endovascular, Curitiba, PR, Brasil.; 2 Instituto VESSEL de Aperfeiçoamento Endovascular de Curitiba, Curitiba, PR, Brasil.; 3 Hospital Nossa Senhora das Graças – HNSG, Serviço de Cirurgia Vascular e Endovascular Elias Abrão, Curitiba, PR, Brasil.; 4 Pontifícia Universidade Católica do Paraná – PUC-PR, Hospital Universitário Cajuru – HUC, Liga Acadêmica de Medicina Vascular – LAMEV, Curitiba, PR, Brasil.

**Keywords:** subclavian artery, thoracic outlet syndrome, critical ischemia

## Abstract

Compression of the subclavian artery in the thoracic outlet is a well-known phenomenon. In rare cases, bone abnormalities, such as pseudarthrosis of the clavicle, can cause arterial compression at this level. Pseudarthrosis may develop as a result of trauma, which is the more common form, or it may be congenital. Here, the authors describe the case of a 44-year-old patient with critical ischemia of the right upper limb. She had a history of untreated right clavicle fracture at 9 months of age which had progressed to pseudarthrosis and extrinsic compression of the subclavian artery causing occlusion. The segment of the clavicle involved was resected and late thromboembolectomy of the subclavian, brachial, distal arteries was performed, with good results.

## INTRODUCTION

 Pseudarthrosis of the clavicle may be congenital or acquired. Acquired or posttraumatic pseudarthrosis is more common and is related to fracture of the clavicle. 1 ^,^ 2 The location of the clavicle means that a pseudarthrosis may cause compression of structures in the thoracic outlet. Compression of the subclavian artery is rare 3 ^-^ 6 and when it occurs it is the result of bone disorders in 88% of cases. 5 The symptoms are generally variable and slow to appear. 7 In this article, the authors report the case of a patient with critical ischemia of the right upper limb caused by chronic compression of the subclavian artery by pseudarthrosis of the clavicle. 

## CASE DESCRIPTION

 The patient was a 44-year-old female craftswoman who had been suffering pain in the right upper limb for several months. The pain had increased progressively over the previous 3 months. She described pain at rest associated with coldness, pallor, and paresthesia of the limb. She had fallen from stairs when 9 months old, fracturing her clavicle, which was managed conservatively. 

**Figure 1:**
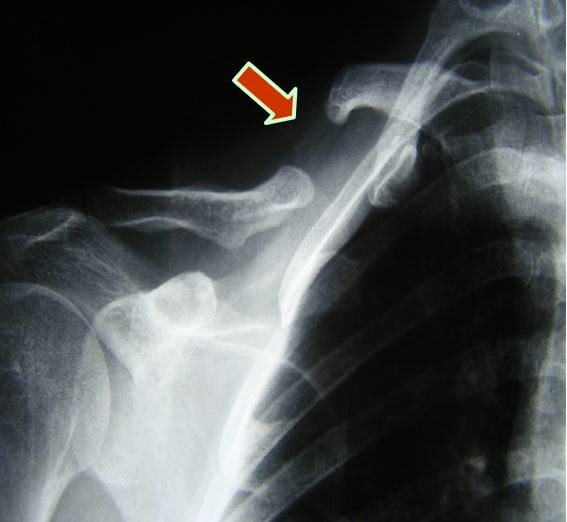
Chest X-ray showing pseudarthrosis of the right clavicle.

 Physical examination revealed discrete deformity at the level of the mid third of the right clavicle. The right hand was cold and blue and ulnar, radial, brachial, and axillary pulses were all absent. Continuous wave Doppler detected no blood flow distally and monophasic flow in the brachial and axillary arteries. The chest X-ray showed a deformity of the mid third of the right clavicle, compatible with pseudarthrosis ( Figure 1 ). 

**Figure 2:**
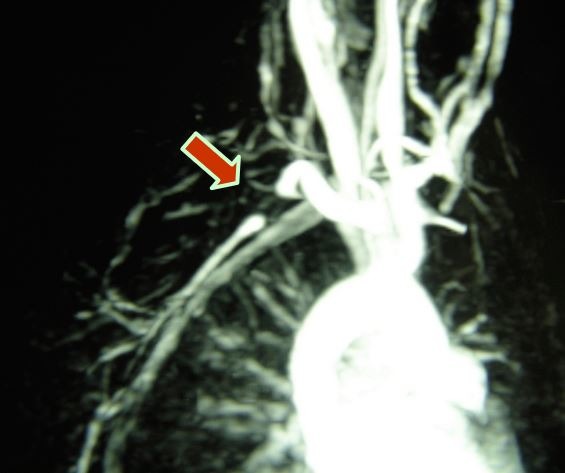
Magnetic resonance angiography showing occlusion of the right subclavian artery.

 Doppler ultrasonography demonstrated segmental occlusion of the subclavian artery with distal refilling via collateral vessels and very fine, threadlike flow in the axillary artery with occlusion of the brachial and radial arteries and refilling of the distal ulnar artery. Magnetic resonance angiography confirmed the Doppler ultrasonography findings ( Figure 2 ). 

**Figure 3:**
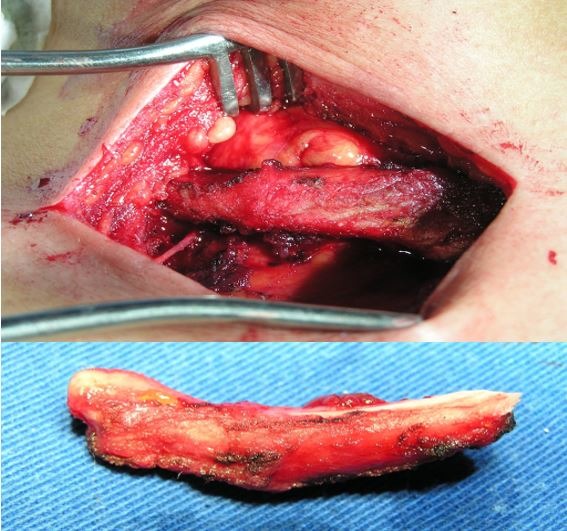
Details of bone resection.

 The pseudarthrosis was treated surgically, via a right supraclavicular incision, with resection of the mid segment of the clavicle ( Figure 3 ). The right subclavian artery was compressed and had thrombi with a chronic appearance inside. Thromboembolectomy of the subclavian artery was conducted with a 3F Fogarty catheter, followed by closure of the arteriotomy with a patch harvested from the saphenous vein in the thigh. Thromboembolectomy of the brachial, radial, and ulnar arteries was then conducted via an arteriotomy in the brachial artery, resulting in considerable improvement in terms of pain, temperature, color, and perfusion of the limb. 

 Throughout the examination, Doppler ultrasound of the hand showed triphasic flow in the radial and ulnar arteries. The patient was kept on clinical anticoagulant treatment with warfarin, maintaining an international normalized ratio between 2.0 and 3.0. Follow-up Doppler ultrasonography was conducted annually for 5 years and the patient attended for clinical follow-up for more than 10 years, until she died from unrelated causes. 

## DISCUSSION

 Compression of the subclavian artery in the thoracic outlet can be caused by bone deformities at this level, such as pseudarthrosis of the clavicle, cervical rib, hypertrophic bone calluses, and luxations. 8 ^,^ 9 It can also be caused by repetitive use of the upper limb, as occurs in some professional and sporting activities. 3

 Arterial complications caused by post-traumatic pseudarthrosis are uncommon, 3 ^-^ 7 but can be severe. 8 The mechanism of injury is the result of chronic constriction of the subclavian artery and repeated microtraumas. There are four clinical variants of arterial injury: thrombosis, microemboli, and formation of aneurysms 7 ^,^ 8 and pseudoaneurysms. 10 The most common form is a combination of aneurysm of the subclavian artery and distal embolization. 3 ^,^ 11 The mechanism of injury in the case described here was probably a combination of arterial thrombosis and distal emboli. 

 In these cases, care should be taken when diagnosing arterial involvement, since arterial compression can be a frequent and incidental finding in people who do not have bone deformities. Compression of the subclavian artery at the level of the thoracic outlet is common in the general population, during compression maneuvers at the level of the interscalene triangle and the costoclavicular space, but the great majority of these individuals are asymptomatic. 12 Constant arterial compression, irrespective of maneuvers and positioning of the limb, in conjunction with bone deformities suggests a causal relationship between the two. In these situations, color Doppler ultrasonography is an excellent screening examination, and should be the first test ordered, since it is noninvasive and inexpensive. Another advantage is the ability to rule out other possible causes of compression and perform maneuvers to induce dynamic compression. 7 ^,^ 13 Angiotomography is a valid option for assessment in this type of pathology, because it shows the relationships between vessels and their adjacent structures. However, exposure to radiation and the need for iodinated contrast restricts its use with certain types of patients, such as in the present case. Although magnetic resonance angiography has inferior image definition compared to angiotomography, it can provide reliable information on vessels in the chest as far as the brachial artery region. More distal arteries of the forearm and the hand are better evaluated using digital arteriography, particularly if there is a suspicion of distal emboli. 14 ^,^ 15

 In these cases, treatment consists of revascularization of the extremity affected and removal of the cause of compression. Treatment with open vascular surgery should therefore be considered the first management option, since the open incision is needed for removal of the bone deformity. 7 ^,^ 8 Endovascular treatments, such as, for example, pharmacological or pharmacomechanical thrombolysis, are of questionable value in these cases. Even if the thrombolytic treatment achieves satisfactory results in terms of dissolving acute and subacute thrombi, the underlying arterial injury should only be treated after removal of the bone deformity. Fitting a stent to treat the original arterial injury without removing the factor causing compression is totally contraindicated, because of the possibility of compression of the stent, fracture of its metal mesh, and consequent stent thrombosis. 7

 The surgical access utilized in the case reported here was a supraclavicular incision. This access is often used to treat thoracic outlet syndrome since it offers sufficient surgical exposure and is satisfactory for the majority of patients who require subclavian artery repair. 16

 Based on experience with surgical treatment of thoracic outlet syndrome, the results of this type of surgery to relieve arterial compression are generally good. However, the results may not be satisfactory if there is neurological involvement because of compression of the brachial plexus for prolonged periods and symptoms can worsen due to the possibility of irreversible nerve damage. 8

 It is therefore essential that a detailed history and complete physical examination are conducted in cases of ischemia with atypical presentation, as in the case of the patient described here, to enable recognition of rare causes of compression and subsequent treatment. 
