# Supplementary material for: Late presentation of critical upper limb ischemia caused by pseudarthrosis of the clavicle
Source: J Vasc Bras. 2018 Apr-Jun;17(2):174–7. doi: 10.1590/1677-5449.009617 (PMC6205708; doi:10.1590/1677-5449.009617)
Supplement: Translation

# Translation: Compressão da artéria subclávia por pseudoartrose de clavícula: apresentação na quinta década de vida

**Authors:** Marcio Miyamotto, Lucas Vasconcelos Sanvido, Luan Facttore Brendolan, Amilton Cezar, Giana Caroline Strack Neves, Izara Castro de Souza, Ricardo César Rocha Moreira

**Affiliations:** 1 Pontifícia Universidade Católica do Paraná – PUC-PR, Hospital Universitário Cajuru – HUC, Serviço de Cirurgia Vascular e Endovascular, Curitiba, PR, Brasil.; 2 Instituto VESSEL de Aperfeiçoamento Endovascular de Curitiba, Curitiba, PR, Brasil.; 3 Hospital Nossa Senhora das Graças – HNSG, Serviço de Cirurgia Vascular e Endovascular Elias Abrão, Curitiba, PR, Brasil.; 4 Pontifícia Universidade Católica do Paraná – PUC-PR, Hospital Universitário Cajuru – HUC, Liga Acadêmica de Medicina Vascular – LAMEV, Curitiba, PR, Brasil.

**Keywords:** artéria subclávia, síndrome do desfiladeiro torácico, isquemia crítica

## Abstract

A compressão da artéria subclávia no desfiladeiro torácico é um fenômeno amplamente conhecido. Anormalidades ósseas, como a pseudoartrose da clavícula, podem raramente causar compressão arterial a esse nível. A pseudoartrose pode desenvolver-se em decorrência de um trauma, que é a forma mais comum, ou ser congênita. Os autores descrevem o caso de uma paciente de 44 anos com quadro de isquemia crítica de membro superior direito. Apresentava história de fratura não tratada de clavícula direita aos 9 meses de idade que evoluiu com pseudoartrose e compressão extrínseca com oclusão da artéria subclávia. O segmento da clavicula acometido pela pseudoartrose foi ressecado e realizada uma tromboembolectomia tardia das artérias subclávia, braquial e distais, com boa evolução.

## INTRODUÇÃO

 A pseudoartrose de clavícula pode ser congênita ou adquirida. A pseudoartrose adquirida ou pós-traumática é mais comum e está relacionada a fraturas de clavícula 1 ^,^ 2 . Devido à sua localização, a pseudoartrose de clavícula pode causar compressão das estruturas do desfiladeiro torácico. A compressão da artéria subclávia é incomum 3 ^-^ 6 e ocorre pela presença de anormalidades ósseas em 88% dos casos 5 . Os sintomas geralmente são variáveis e de aparecimento tardio 7 . Os autores relatam o caso de uma paciente com isquemia crítica de membro superior direito causada pela compressão crônica da artéria subclávia por pseudoartrose de clavícula. 

## DESCRIÇÃO DO CASO

 Paciente do sexo feminino, artesã de 44 anos, apresentava história de dor em membro superior direito havia vários meses. Referiu aumento progressivo da dor nos últimos 3 meses, evoluindo para dor ao repouso associada a esfriamento do membro, palidez e parestesia. Apresentava história prévia de queda da escada com 9 meses de idade, com fratura de clavícula manejada de forma conservadora. 

 No exame físico, apresentava deformidade discreta ao nível do terço médio da clavícula direita. A mão direita apresentava-se fria e cianótica, com ausência de pulsos ulnar, radial, braquial e axilar. O fluxo ao Doppler de onda contínua era ausente distalmente e monofásico nas artérias braquial e axilar. O raio X de tórax mostrou deformidade no terço médio da clavícula direita, compatível com pseudoartrose ( Figura 1 ). 

**Figura 1:** Raio x de tórax evidenciando pseudoartrose da clavícula direita.

 O eco-Doppler demonstrou oclusão segmentar da artéria subclávia com reenchimento distal por colaterais, além de fluxo filiforme na artéria axilar com oclusão das artérias braquial e radial e reenchimento na artéria ulnar distal. A angiorressonância confirmou os achados do eco-Doppler ( Figura 2 ). 

**Figura 2:** Angiorressonância evidenciando oclusão da artéria subclávia direita.

 O tratamento cirúrgico da pseudoartrose foi realizado através de uma incisão supraclavicular direita para ressecção do segmento médio da clavícula ( Figura 3 ). A artéria subclávia direita apresentava-se comprimida e com trombos de aparência antiga no seu interior. Foi realizada tromboembolectomia com cateter de Fogarty 3F da artéria subclávia e síntese da arteriotomia com remendo de veia safena da coxa. A seguir realizou-se tromboembolectomia das artérias braquial, radial e ulnar através de arteriotomia da artéria braquial, com melhora importante da dor, da temperatura, da coloração e da perfusão do membro. 

**Figura 3:** Detalhes da ressecção óssea.

 A paciente permaneceu, durante o exame, com fluxo trifásico nas artérias radial e ulnar ao Doppler de mão. Foi mantida em tratamento clínico anticoagulante com warfarina, mantendo-se uma razão normatizada internacional (RNI) entre 2,0 e 3,0. Permaneceu em seguimento clínico por mais de 10 anos, até seu óbito por causa não relacionada, sendo realizado eco-Doppler anual de seguimento por 5 anos. 

## DISCUSSÃO

 A compressão da artéria subclávia no desfiladeiro torácico pode ser causada por deformidades ósseas a esse nível, como pseudoartrose de clavícula, costela cervical, calos ósseos hipertróficos e luxações 8 ^,^ 9 . Também pode ser causada por esforço repetitivo do membro superior, como ocorre em algumas atividades profissionais e esportivas 3 . 

 As complicações arteriais decorrentes de pseudoartrose pós-traumática são infrequentes 3 ^-^ 7 , e podem ser graves 8 . O mecanismo de lesão é decorrente da constrição crônica da artéria subclávia e microtraumatismos de repetição. Há quatro variantes clínicas da lesão arterial: trombose, microembolia, formação de aneurisma 7 ^,^ 8 e pseudoaneurisma 10 . A forma mais encontrada é a associação de aneurisma da artéria subclávia com embolização distal 3 ^,^ 11 . O provável mecanismo de lesão no caso aqui descrito foi uma associação de trombose arterial e embolia distal. 

 Para o diagnóstico de envolvimento arterial, nesses casos é necessário ter cautela, pois a compressão arterial pode ser um achado freqüente e incidental em pessoas que não apresentam deformidades ósseas. A compressão da artéria subclávia é comum ao nível do desfiladeiro torácico na população geral, durante as manobras de compressão ao nível do triângulo escalênico e do espaço costoclavicular, sendo que a grande maioria desses indivíduos é assintomática 12 . A compressão arterial fixa, independentemente de manobras e do posicionamento do membro na presença de deformidades ósseas, sugere relação de nexo causal entre as duas entidades. Nessas situações, o eco-Doppler colorido é um excelente exame de rastreio, e deve ser o primeiro exame a ser solicitado, por ser não invasivo e de baixo custo. Outra vantagem é a possibilidade de descartar outras possíveis causas de compressão e também de realizar manobras de indução em compressões dinâmicas 7 ^,^ 13 . A angiotomografia é uma opção válida para avaliação nesse tipo de patologia, pois mostra a relação entre os vasos e as estruturas adjacentes. A exposição à radiação e a necessidade do uso de contraste iodado restringe sua utilização em determinados tipos de pacientes, como no presente caso. A angiorressonância, embora apresente uma definição de imagem inferior à da angiotomografia, oferece informações confiáveis em vasos do tórax até a região da artéria braquial. Artérias mais distais do antebraço e da mão são mais bem avaliadas através da arteriografia digital, principalmente quando existe a suspeita de embolia distal 14 ^,^ 15 . 

 O tratamento nesses casos é a revascularização da extremidade afetada e a retirada do fator compressivo. Sendo assim, o tratamento através de cirurgia vascular aberta deve ser considerado como primeira opção de abordagem, já que há a necessidade de incisão aberta para a retirada da deformidade óssea 7 ^,^ 8 . O tratamento endovascular, como por exemplo a trombólise farmacológica ou farmacomecânica, é uma alternativa discutível nesses casos. Mesmo que haja um resultado satisfatório com o tratamento trombolítico na dissolução dos trombos agudos e subagudos, a lesão arterial subjacente deverá ser tratada somente após a remoção da deformidade óssea. O implante de um stent para tratar a lesão arterial básica sem a retirada do fator compressivo é totalmente contraindicado, devido à possibilidade de compressão do stent, fratura da malha metálica e consequente trombose do stent 7 . 

 O acesso cirúrgico utilizado no caso aqui relatado foi o acesso supraclavicular. Esse acesso, realizado com frequência para o tratamento da síndrome do desfiladeiro torácico, oferece uma exposição cirúrgica suficiente e é satisfatório na maioria dos pacientes que necessitam de reparo da artéria subclávia 16 . 

 Baseando-se na experiência com o tratamento cirúrgico da síndrome do desfiladeiro torácico, esse tipo de cirurgia para descompressão arterial geralmente apresenta bons resultados. Quando há comprometimento neurológico pela compressão do plexo braquial por um período de tempo prolongado, os resultados podem não ser satisfatórios, considerando a piora dos sintomas devido à possibilidade de lesões irreversíveis do nervo 8 . 

 Desse modo, é imprescindível a realização de uma anamnese detalhada e um exame físico completo em casos de isquemia de apresentação atípica como o da paciente aqui apresentada, nos quais fatores compressivos raros podem ser reconhecidos e corretamente tratados.
